# Cardiac Tamponade in a Suspected COVID-19 Patient

**DOI:** 10.7759/cureus.85788

**Published:** 2025-06-11

**Authors:** Gurpreet Singh

**Affiliations:** 1 Medicine, Palomar Medical Center, Escondido, USA

**Keywords:** cardiac tamponade, covid-19, multifocal pneumonia, pcr test, shortness of breath (sob)

## Abstract

A 59-year-old male with no significant medical history presented with dyspnea and bilateral opacities on chest X-ray. The patient had a high suspicion for COVID-19 pneumonia based on chest X-ray and laboratory findings. Echocardiogram was performed, and the patient was found to have a large pericardial effusion with tamponade. An emergent pericardiocentesis was performed, with 600 cc of bloody fluid removed.

## Introduction

Since late December 2019, there has been an outbreak of a novel enveloped RNA betacoronavirus called severe acute respiratory syndrome coronavirus 2 (SARS-CoV-2) [1]. This virus causes coronavirus disease 2019 (COVID-19), which has become an ongoing pandemic. The novel SARS-CoV-2 is the seventh member of the Coronaviridae family known to infect humans. The clinical course of SARS-CoV-2 infection is mostly characterized by respiratory tract symptoms, including fever, cough, pharyngodynia, fatigue, and complications related to pneumonia and acute respiratory distress syndrome [2]. We report an important case in which cardiac tamponade was diagnosed in a patient with a high clinical suspicion for COVID-19, which eventually led to his death. The patient had a negative diagnostic test; however, clinical suspicion remained very high, as this case occurred in April 2020, the prevalence of COVID-19 was very high, and diagnostic tests were less sensitive than what is currently available. There have been a few cases reported with COVID-19-associated cardiac tamponade [3]. Cardiac tamponade may be a potential complication of COVID-19 infection that should be taken into consideration. Delayed or missed diagnosis can have dire consequences for the patient.

## Case presentation

A 59-year-old man with no previous medical history presented to the emergency room (ER) with acutely worsening dyspnea. He was in his usual state of health before developing a hacking dry cough with worsening dyspnea on exertion over the course of four weeks. He also reported decreased oral intake with profound nausea and fatigue the week before arrival. The patient denied any chest pain, palpitations, lightheadedness, dizziness, diarrhea, fevers, chills, or any other associated complaints. The patient denied any recent travel or sick contacts; however, he worked as a waiter in New York City. The patient was noted to have tachycardia at 101 beats/minute and blood pressure of 152/71 mmHg. He was afebrile and saturating at 85% on room air, which improved to 95% on 2 L of oxygen via nasal cannula. Physical examination at the time of presentation was significant for elevated jugular venous distension about 4 cm, bilateral lower extremity pitting edema, and decreased breath sounds with basilar crackles. The rest of his examination was otherwise unremarkable. Given the aforementioned findings, the patient was admitted for further workup and management of suspected COVID-19 viral pneumonia.

Investigations

Table 1 presents the laboratory findings of the patient at the time of admission.

**Table 1:** Laboratory findings. CRP = C-reactive protein; pBNP = pro-B-type natriuretic peptide; CKMB = creatine kinase myocardial band; PCR = polymerase chain reaction

Serum laboratory test	Value	Reference range
Creatinine phosphokinase	1,081 U/L	35–232 U/L
Ferritin	2,776 ng/mL	26–388 ng/mL
CRP	70 mg/L	<3 mg/L
White blood cell count	17 K/µL	4–10 K/µL
Lymphocyte	6%	15–47%
pBNP	930 pg/mL	<450 pg/mL
Creatinine	1.74 mg/dL	0.95 mg/dL
CKMB	Undetectable	<4.4 ng/mL
troponin I	Undetectable	<0.04 ng/mL
COVID-19 PCR nasal swab	Negative	

**Figure 1:**
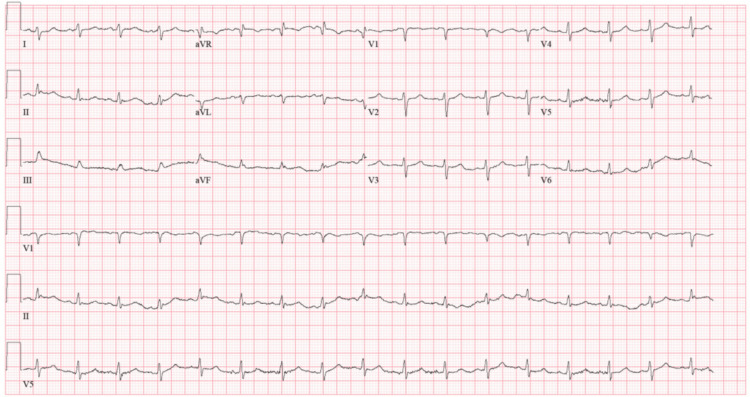
Electrocardiogram showing sinus tachycardia with low voltage.

The patient’s electrocardiogram (ECG) (Figure 1) confirmed sinus tachycardia with low voltage and no obvious ischemic changes.

**Figure 2:**
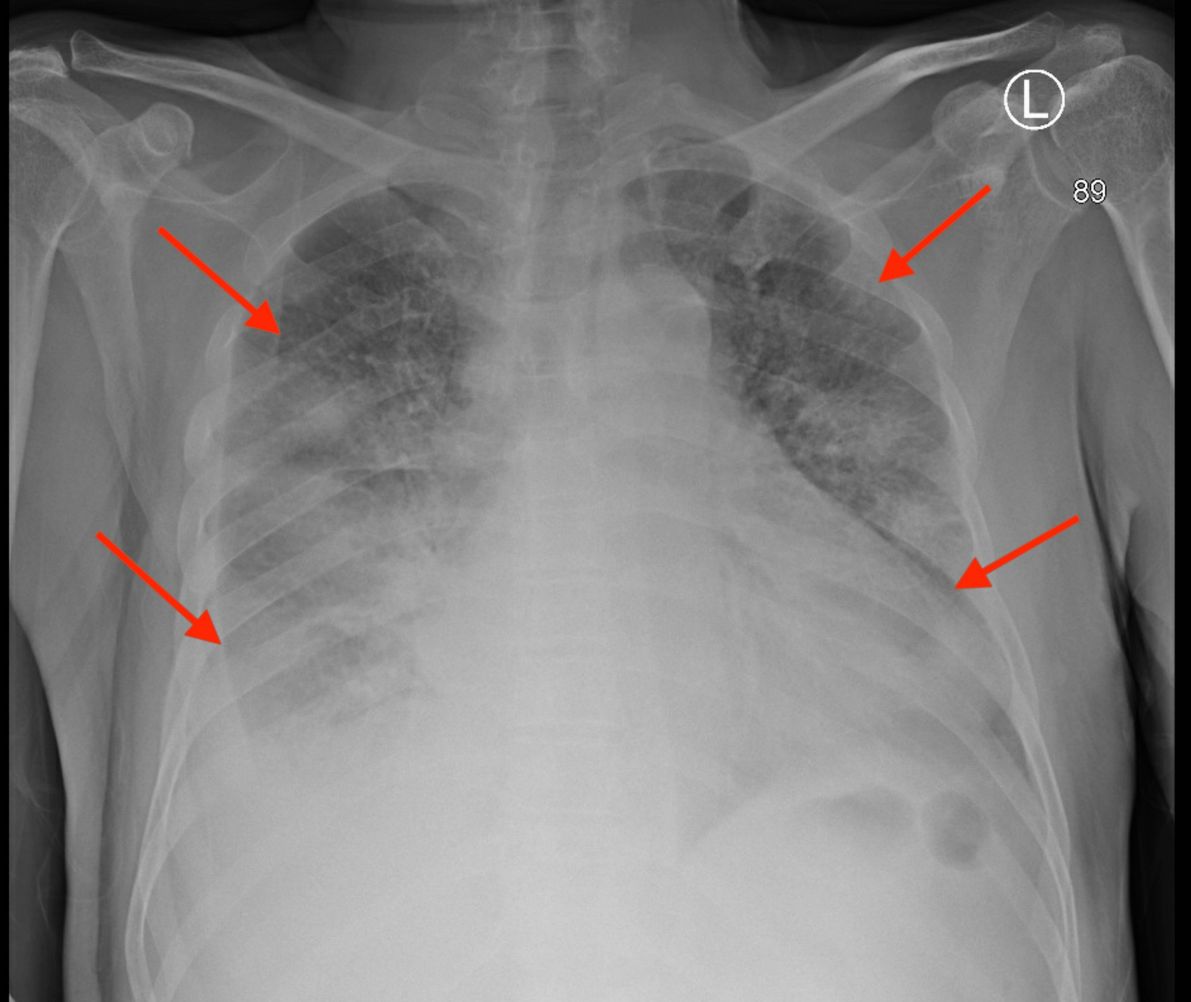
Chest X-ray revealed bilateral peripheral and basilar infiltrates along with cardiomegaly.

Chest X-ray (CXR) (Figure 2) revealed bilateral peripheral and basilar infiltrates along with cardiomegaly.

**Figure 3:**
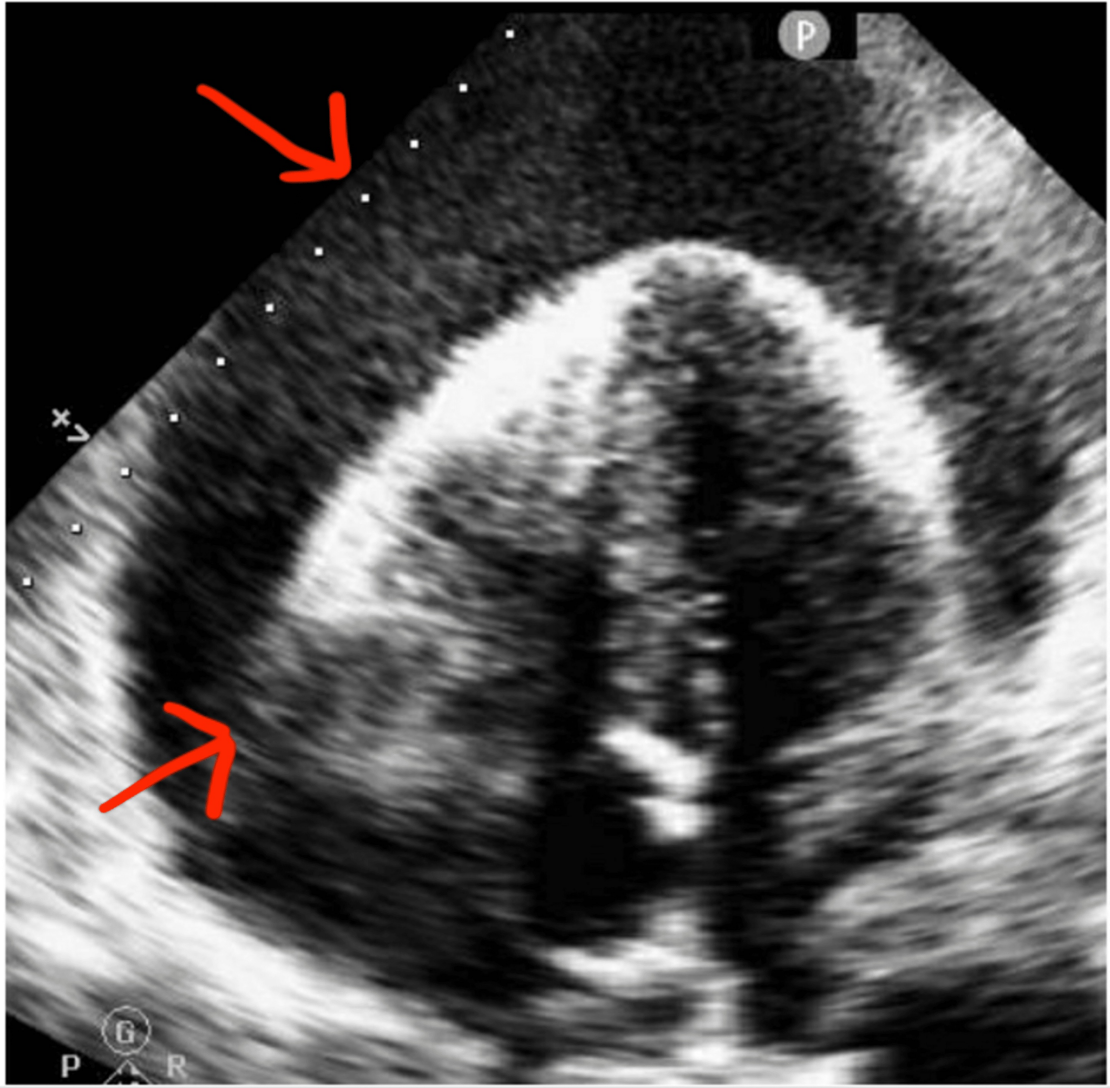
Echocardiogram showing large circumferential effusion up to 4.9 cm with right ventricular collapse.

Echocardiogram was performed showing an ejection fraction of 50-55% and large circumferential effusion up to 4.9 cm with right ventricular (RV) collapse (Figure 3).

Management

Blood pressure, heart rate, and oxygen requirements remained stable. Given the patient’s bilateral opacities on CXR with elevated C-reactive protein (CRP), lymphopenia, elevated ferritin, and creatine phosphokinase (CPK), there was a high suspicion for COVID-19 pneumonia. This case presentation occurred in April 2020 when COVID-19 infection was highly prevalent. Bacterial pneumonia was not ruled out, and the patient was placed on treatment for community-acquired pneumonia with doxycycline and ceftriaxone along with isolation precautions. Other differentials included new acute congestive heart failure given elevated pro-B-type natriuretic peptide, shortness of breath, and leg swelling. Echocardiogram was performed on hospital day two, revealing a large cardiac tamponade. Cardiology was consulted, and the patient was taken for urgent pericardiocentesis. The patient’s vitals continued to remain stable. Upon arrival at the catheterization laboratory, the patient went into cardiac arrest, and cardiopulmonary resuscitation (CPR) was performed with endotracheal intubation. Emergency pericardiocentesis was performed while CPR continued. More than 600 cc of bloody fluid removed. Return of spontaneous circulation (ROSC) was achieved, and the patient was started on dobutamine and norepinephrine. Follow-up echocardiogram showed resolution of effusion and expansion of the RV and right atrium. Despite resuscitative efforts and vasopressors, the patient died within two hours after ROSC was achieved.

## Discussion

COVID-19 was discovered in Hubei Province, China, in December 2019 [1]. A cluster of patients was admitted with fever, cough, shortness of breath, and other symptoms [1]. There have been a few case reports of cardiac involvement with COVID-19, including cardiac tamponade, cardiac effusion, pericarditis, myocarditis, and myocardial infarction [3,4]. While this patient’s initial COVID-19 swab was negative, there has been a significant number of false negatives on the initial swab [5]. False-negative rates for someone who had symptoms more than 10 days ago are nearly 33% using a nasal swab and 52.89% for a throat swab [6]. A specimen collection at the wrong time almost certainly contributes to false-negative test results [4]. If the sample cannot be sent immediately, it can be refrigerated at 2-8°C for up to 72 hours. If transport is not possible within 72 hours, then the sample should be stored at -70°C or below. Thus, if a specimen is not transported or stored appropriately, the risk of a false-negative reverse transcription-polymerase chain reaction result increases. Site of collection also matters; oropharyngeal swabs detected SARS-CoV-2 RNA in only 32% of swabs, which was significantly lower than the level in nasal swabs (63%) [7].

Despite a negative COVID-19 nasal swab test, this patient’s suspicion for COVID-19 pneumonia remained high as his CXR had bilateral infiltrates along with laboratory findings of lymphopenia, elevated CRP, ferritin, and CPK levels, with plans for re-swab and serology testing. However, re-swabbing was not done as the patient expired. This patient’s symptoms had been ongoing for three to four weeks, which may have resulted in a higher chance of false-negative results on the COVID-19 swab. This patient had no significant past medical history and developed a large pericardial hemorrhagic effusion. There were no signs of direct myocyte damage as his troponin I levels and creatine kinase myocardial band were negative without signs of ischemic changes on ECG, thus making myocarditis and acute coronary symptoms less likely.

It is unclear which pathophysiologic mechanism led to the effusion. Inflammation may make the intercalated disks between myocytes to become leaky, resulting in an effusion. COVID-19 causes significant inflammation in the body, resulting in high CRP, ferritin, CPK, and lactate dehydrogenase, as seen in this patient [8]. There have been reports of viral cardiac effusions with viruses such as the Epstein-Barr virus, influenza A, echovirus, HIV, and coxsackie B [9], and now we have cases associated with COVID-19 [3].

Pericardial effusion results from the accumulation of fluid in the pericardial sac, which may be transudative, exudative, or sanguineous and may contain infectious organisms or malignant cells. Inflammation, infection, dissection, or trauma are the most common etiologies. This patient did not have any traumatic injuries or signs of aortic dissection. It was likely due to inflammation or possibly direct viral injury. COVID-19 has been reportedly found in pericardial effusion by PCR analysis, possibly suggesting viral tropism in cardiac tissue [10]. Unfortunately, this patient’s pericardial fluid was not sent for analysis to evaluate for malignancy or infectious causes.

## Conclusions

Inflammation secondary to viral infection, such as COVID-19, may have extrapulmonary complications, such as cardiac tamponade, which may be missed. Physical examination remains an important tool in diagnosis, as echocardiograms may not be readily available. Although COVID-19 was not confirmed in this patient, a negative initial swab for COVID-19 may not rule out infection. Laboratory and clinical picture should be taken into account, as swab false-negative results can be affected by the time of collection and storage.
